# Long-term survival and successful conservation? Low genetic diversity but no evidence for reduced reproductive success at the north-westernmost range edge of *Poa badensis* (Poaceae) in Central Europe

**DOI:** 10.1007/s10531-019-01722-x

**Published:** 2019-02-27

**Authors:** Kristina Plenk, Katharina Bardy, Maria Höhn, Matthias Kropf

**Affiliations:** 1 Institute for Integrative Nature Conservation Research, University of Natural Resources and Life Sciences, Vienna, Gregor-Mendel-Str. 33, 1180 Vienna, Austria; 2 Department of Botany, Faculty of Horticultural Science, Szent István University, Ménesi Str. 44, 1118 Budapest, Hungary

**Keywords:** Peripheral populations, Transect, AFLPs, cpDNA sequence variation, Germination experiments, Conservation history

## Abstract

Many steppe species reach their (north)westernmost distribution limit in western Central Europe. This also applies to *Poa badensis*, a rare steppe plant of calcareous rock/sand vegetation. To explore potential differences in reproductive success and genetic composition of peripheral populations, we analysed the absolute (north)westernmost occurrences in Western Germany and populations at the western margin (Eastern Austria) and the centre (Central Hungary) of the Pannonicum, representing a part of the continuous range. Specifically, we discuss the genetic and reproductive constitution of the (north)westernmost exclave and draw conclusions on the species’ biogeographical and conservation history in this region. Therefore, we used two independent molecular marker systems (AFLPs, cpDNA sequences) and a set of performance parameters. Overall, lowest regional genetic diversity was found in Western Germany, which is mainly a result of the specific history of two populations. However, this low genetic diversity was not accompanied by reduced reproductive success. The Eastern Austrian populations showed reduced genetic diversity and predominantly reduced performance, interpreted as a consequence of small population sizes. Central Hungarian populations showed the overall highest genetic diversity and comparatively high performance values. We observed high admixture and haplotype sharing between Austrian and Hungarian populations, indicating gene flow among these regions. In contrast, we interpreted the increased population differentiation within, and the clear distinctiveness of the German exclave as a long-term isolation of these (north)westernmost occurrences. Our results, overall, prove the good constitution of these populations and, together with their particular biogeographical history, highlight their conservation value.

**Electronic supplementary material:**

The online version of this article (10.1007/s10531-019-01722-x) contains supplementary material, which is available to authorized users.

## Introduction

Investigating the constitution of (rare) species at their absolute distributional periphery, we can test commonly and controversially discussed biogeographical hypotheses, e.g. the ‘abundant centre’ distribution (Sagarin and Gaines 2002) or the ‘central-marginal hypothesis’ (Eckert et al. 2008; see also Sexton et al. 2009; Abeli et al. 2014; Pironon et al. 2016). As a result of more unfavourable conditions at the absolute distribution limit, peripheral populations are generally assumed to have smaller population sizes, occur in lower densities and be genetically more differentiated among each other, compared to equivalents in more central parts of the distribution range. Subsequently, genetic bottlenecks, genetic drift and inbreeding may reduce genetic diversity and influence species’ fitness and potential for adaptation to local conditions. These predictions on the genetic composition were partially detected in several studies on steppe plant species (cf. Pérez-Collazos et al. 2009; Wagner et al. 2011a; but see also Kajtoch et al. 2016). However, few studies have analysed the influence on species’ performance and the expected patterns of reduced reproductive success were mostly not observed (cf. Wagner et al. 2011b; but see also Pironon et al. 2016).

Nevertheless, isolated peripheral populations are not necessarily characterised by such parallel patterns. Besides hypotheses on centrality-marginality phenomena, the existence of extrazonal, small-scale refugia, persisting in situ during the Quaternary or even longer (e.g. Plenk et al. 2017), must also be considered. In Central Europe, where steppe species today reach their absolute westernmost distribution limit (cf. Meusel 1970; Lang 1994), such extrazonal refugia can be found north of the Alps, in the Carpathians and the Pannonian region. Depending on the variability of the ancestral gene pool, as well as the probability of and time for accumulation of de novo mutations and/or effects of lineage sorting (Avise 2000), these relict populations could hold comparatively high levels of unique genetic diversity (cf. Schmitt and Varga 2012; Kajtoch et al. 2016; Plenk et al. 2017). The increasing isolation at the distributional periphery and the subsequently increasing genetic differentiation of populations due to genetic drift may have additionally led to high levels of regional genetic diversity; characteristics similar to those described by Hampe and Petit (2005) for ‘stable’ rear edge populations.

**Fig. 1:**
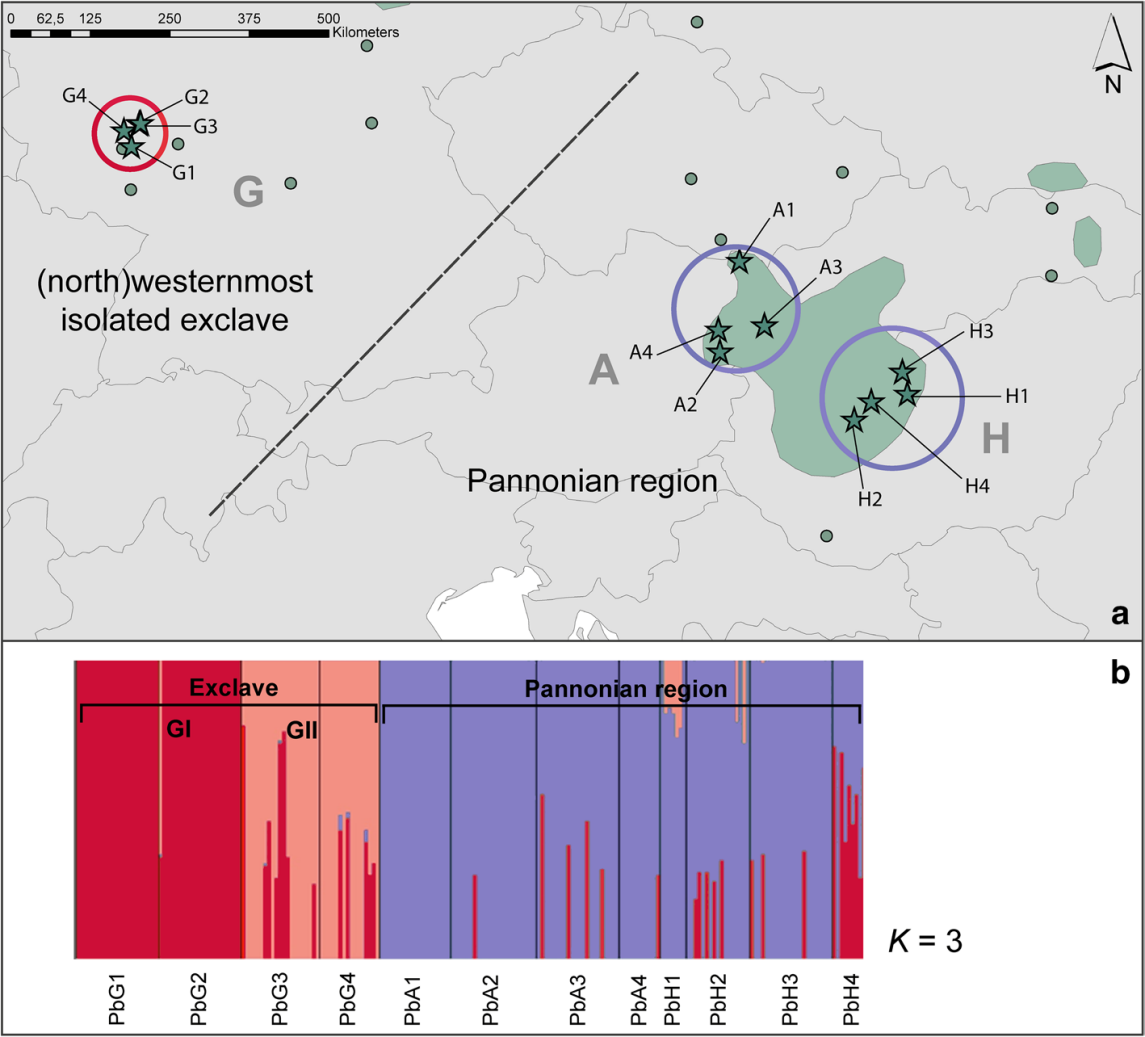
Sampling, distribution and admixture of *Poa badensis.* Distribution of *P. badensis* along our transect (**a**), representing the three study regions (circles) and sampled populations (stars). The species’ continuous Central European distribution is expressed in full-colour, disjunct occurrences as coloured points (distribution map based on Meusel et al. 1965). AFLP-based proportions of admixture (**b**) derived from Bayesian spatial clustering of individuals (*K *= 3). For population abbreviations see Table 1

*Poa badensis* is a perennial, thermophilous grass with a disjunct submediterranean distribution mainly restricted to Central and Southeastern Europe (cf. Figure 1; Meusel et al. 1965), as well as to the southeast of France (Conert 1998). Beside this, there are probably further occurrences in northern Anatolia (cf. Buschmann 1942) and the Caucasus (cf. Conert 1998; Zernov et al. 2015, where *P. alpina* “incl. *P. badensis”* is listed). At its (north)westernmost distribution limit the species grows at isolated sites in Central and Western Germany (cf. Blaufuss and Reichert 1992; Hensen and Wesche 2007; Hodvina and Cezanne 2008). These scattered occurrences are considered to be relict localities of a former (postglacially) more widely distributed steppe complex (Meusel 1970; Bredenkamp et al. 2002) and often have a specific conservation history.

However, the phylogeography of *Poa badensis* is mostly unknown. The only available genetic study was based on AFLPs and included Central German populations from Saxony-Anhalt and Thuringia, exclusively (Hensen and Wesche 2007). Hensen and Müller (1997) investigated the anemochorous dispersal ability of *P. badensis*, however, reproductive fitness in wild populations of our study species has not been investigated, previously. Combined studies on performance *and* genetic characterisation of steppe species are rare (and lacking for *P. badensis*) and concentrated mostly at a regional scale (cf. Hensen et al. 2005; Dostálek et al. 2010; Lauterbach et al. 2011; Heinicke et al. 2016), with the exception of the work on *Stipa capillata* by Wagner et al. (2011a, b).

In this study on *P. badensis,* we focused on the (north)westernmost distribution of this thermophilous (rock) steppe species (cf. Willner et al. 2017) and explored differently peripheral populations within its comparatively narrow Central European distribution range. We included representative populations from the absolute periphery as well as from the margins of its continuous Pannonian distribution range, covering three different study regions. To analyse the genetic structure and diversity of *P. badensis* along this study transect, we used two molecular marker systems, i.e. Amplified fragment length polymorphisms (AFLPs) and chloroplast (cpDNA) sequence data. We also tested the reproductive success of this rare species across the same populations by estimating different performance parameters in a germination experiment. Based on these datasets, we evaluated the predicted general characteristics of peripheral populations. Specifically, we aimed at answering the following questions and finally draw conclusions on the species’ history in the German exclave: (1) Can we detect different patterns of genetic composition and reproductive success along our study transect, following predictions on *centrality*-*marginality*? Or do the patterns rather reflect a particular history of the three study regions, indicating long-term (extrazonal) refugia at the edge of the species’ distribution? (2) Do genetic and performance parameters vary in parallel: are populations of the (north)westernmost exclave less viable, i.e. showing decreased genetic diversity, highly (genetically) isolated populations *and* reduced reproductive success? Finally (3) we conclude what can be deduced from our results with respect to the species’ biogeographical and conservation history in the (north)westernmost exclave in Central Europe.

## Materials and methods

In a statistical large-scale re-classification of Pannonian and Western Pontic steppe grasslands, Willner et al. (2017) defined *Poa badensis* as a diagnostic species of the order *Stipo*-*Festucetalia pallentis* (i.e. rocky steppes; cf. also Borhidi et al. 2012). Accordingly, the species grows on calcareous grus and rock communities, or more rarely on basic substrates, but can also be found on calcareous sand.

*P. badensis* is wind-pollinated, producing seeds which are dispersed by wind over short distances (Hensen and Müller 1997) and germinate rapidly after release (i.e. with no signs of dormancy; Hensen and Wesche 2007; pers. observ.). The grey-green tussocks produce no vegetative stolons, however, sometimes a clonal reproduction seems to be possible due to tussock fragmentation (cf. Conert 1998, Hensen and Wesche 2007; pers. observ.). Flowering of 4-8 cm long panicles lasts from May to July. One ovate, green to violet spikelet contains 3-6(-9) flowers (Conert 1998). *P*. *badensis* is predominantly described as a diploid species (Müntzing and Nygren 1955; Joshi et al. 2017). Nevertheless, the following chromosome numbers have been reported without precise information about the geographical origin: 2n = 14, 28, 42 (Oberdorfer 2001) and 2n = 14, 18, 21, 28 (Conert 2000; including odd numbers). In the Pannonian region in Eastern Austria the species is diploid (Fischer et al. 2008). To test ploidy levels within our dataset, we exemplarily analysed two Austrian and two Hungarian individuals using the CyFlow^®^Space (Partec, Münster, Germany) and the software FlowMax (version 2.3, Partec 2001). We obtained evidence for a likewise diploid level in these Austrian and Hungarian individuals. In contrast, individuals tested from the German region (n = 4) were observed to be tetraploid (cf. Online Resource 1).

Typically, the ploidy level might be reflected in the number of AFLP fragments, amplified per AFLP phenotype, with higher ploidy levels possibly showing higher fragment numbers (Kardolus et al. 1998). In this sense, the distinctiveness of groups might become blurred by differing averaged AFLP fragment numbers (cf. Guo et al. 2005). However, we observed comparable mean fragment numbers with an even lower value for the assumed tetraploid individuals from the German study region (average 66.4 bands per individual), compared to the assumed diploid individuals from the Pannonian region (average 81.1 bands per individual), and clear grouping patterns in both genetic analyses (i.e. distance-based SplitsTree analysis and Bayesian Clustering analysis; see Results). Therefore, we could reject the possible interference of our AFLP analyses by the difference in ploidy levels and analysed data jointly. Similar un-blurred results were obtained in a number of AFLP studies including differing ploidy levels (e.g. Ma et al. 2010; Španiel et al. 2012).

### Study design

Focusing on the Central European distribution of *Poa badensis,* we defined a study transect (cf. Plenk et al. 2017) crossing Central Hungary and the eastern parts of Lower Austria in the east, and reaching the (north)westernmost isolated occurrences of the species in Western Germany (Fig. 1). More specifically, the Austrian and Hungarian populations are situated at the western margin and in the centre of the Pannonicum, where the species can be found scattered in suitable habitats (Niklfeld 2013; Bartha et al. 2015). In contrast, the German study region in Rhineland-Palatinate (Northern Rhinehesse and Lower Nahe region) nowadays only comprise five currently known populations (Blaufuss and Reichert 1992), which are strongly isolated within this (north)westernmost study region. However, in the past *P. badensis* was more common in this region, representing (genetically) likely connected occurrences, i.e. a distinct exclave, outside its continuous Pannonian distribution range.

To obtain an equal sampling across our study transect, we chose four populations per study region, i.e. twelve populations in total. In addition to this main dataset, we sampled material from the fifth population known in the Western German study region, the Mainzer Sand (G5). This population was not included in our main dataset due to the low number of individuals (i.e. four) found there, in our sampling year (2010). For molecular analyses, we collected leaf material from randomly chosen individuals across the respective population. Additionally, the number of flowering individuals was estimated at the same time to obtain population-size categories for each sampled population (see Table 1). Seeds of *P. badensis* were later collected from the same populations (i.e. from 20 individuals per population; again except G5) by taking one, randomly chosen, mature panicle per fruiting plant.

**Table 1:** Genetic diversity of *Poa badensis* in Central Europe

Code^a^	Locality	Est. PSC^b^	N AFLP^c^	*H*_E_ ± SD^d^	*I* ± SD^e^	*PLP* %^f^	*PLP* % [7]^g^	*Br* [7]^h^	N cpDNA^c^	Number of haplotypes	Nucleotide diversity (π) ± SD	Haplotype diversity (*h*) ± SD	Tajima’s D
G1	Eckelsheim	1	22	0.0457 ± 0.1103	0.0755 ± 0.1675	21.43	21.70	1.127	9	1	0	0	0.00 ns
G2	Weilersberg	4	22	0.0479 ± 0.1097	0.0802 ± 0.1675	23.90	24.00	1.134	9	1	0	0	0.00 ns
G3	Heidesheim	3	21	0.1355 ± 0.1732	0.2125 ± 0.2515	49.61	49.70	1.345	9	1	0	0	0.00 ns
G4	Eierfels	2	16	0.1211 ± 0.1660	0.1910 ± 0.2433	44.42	44.70	1.322	8	1	0	0	0.00 ns
**Germany**		**2.5**		**0.0876**	**0.1398**	**34.84**	**35.00**	**1.232**		**2**	**0**	**0**	**0**
A1	Falkensteiner Berg	1	19	0.0949 ± 0.1529	0.1498 ± 0.2283	34.68	34.70	1.249	9	1	0	0	0.00 ns
A2	Eggendorf	1	23	0.0891 ± 0.1490	0.1427 ± 0.2205	36.62	36.90	1.233	7	2	0.00019 ± 0.00026	0.2857 ± 0.1964	− 1.01 ns
A3	Hundsheimer Berg	3	22	0.0961 ± 0.1488	0.1552 ± 0.2217	40.26	40.40	1.256	7	3	0.00051 ± 0.00048	0.5238 ± 0.2086	0.56 ns
A4	Mödling	2	11	0.0823 ± 0.1523	0.1273 ± 0.2251	26.88	26.90	1.222	7	1	0	0	0.00 ns
**Austria**		**1.75**		**0.0906**	**0.1438**	**34.61**	**34.70**	**1.240**		**4**	**0.00018**	**0.2024**	**− 0.11**
H1	Budaörs	3	7	0.1158 ± 0.1730	0.1759 ± 0.2557	33.77	33.80	1.338	7	2	0.00038 ± 0.00040	0.2857 ± 0.1964	− 1.01 ns
H2	Várpalota	3	17	0.1259 ± 0.1580	0.2030 ± 0.2334	50.91	51.00	1.344	9	1	0	0	0.00 ns
H3	Pilisszentkereszt	4	22	0.1408 ± 0.1687	0.2243 ± 0.2443	55.97	56.10	1.366	6	1	0	0	0.00 ns
H4	Csàkvár	3	9	0.0662 ± 0.1312	0.1054 ± 0.1999	23.64	23.60	1.203	7	2	0.00063 ± 0.00056	0.4762 ± 0.1713	0.56 ns
**Hungary**		**3.25**		**0.1122**	**0.1772**	**41.07**	**41.10**	**1.313**		**2**	**0.00025**	**0.1905**	**− 0.11**

Population code, locality and genetic diversity based on AFLP/cpDNA data of twelve *Poa badensis* populations, including regional mean genetic diversity (in bold)

^a^*G* Germany,* A* Austria,* H* Hungary

^b^Estimated population size category (PSC): 1: < 100, 2: 100–200, 3: 200–500, 4: 500–1000 individuals

^c^Number of included individuals

^d^Nei’s gene diversity (Nei 1973)

^e^Shannon’s information index (Shannon and Weaver 1949)

^f^Percentage of polymorphic loci

^g^*PLP* at 5% after rarefaction to the minimum populational sample size of 7

^h^Band richness after rarefaction to 7;* SD* standard deviation,* ns* not significant

### Germination experiment

This experiment was carried out with 20 individuals per population. For each mother plant the number of flowers (shed spikelets were counted to obtain an estimate of the total number of flowers in the panicle) and the number of seeds per panicle were counted. The seeds were distinguished as mature and undeveloped seeds; total seed mass was then determined for all mature seeds per individual panicle. For germination, usually ten seeds per individual mother plant were used, whereby undeveloped, shrunken seeds were excluded. The seeds were placed in petri dishes on a double-layered filter paper following a definite scheme, which allowed tracking each seed, individually. Petri dishes were placed in a climate chamber for 41 days. The settings for light and temperature mimic a day/night cycle with 12 h and 25 °C (with light) and 12 h and 15 °C (dark). The germination process was documented by differentiating four states for each seedling: ‘not germinated’, ‘radicle’ (visible), ‘cotyledon’ (visible), ‘dead’. Individuals, which successfully germinated (i.e. showing breakthrough of the radicle), were transplanted in multi-pot-plates filled with a standardised mixture of plant soil and quartz sand at a ratio of 2 to 1. After 2 months, seedling survival was determined by recording the plants survived in the multi-pot-plates.

### Molecular methods

To isolate total genomic DNA from silica dried leaf material, ca. 20 mg per sample were ground with sterilized glass pellets and processed using the DNeasy™ Plant Mini Extraction Kit (QIAGEN, Hilden, Germany) according to the manufacturer’s protocol.

*Amplified Fragment Length Polymorphisms (AFLPs)* profiles were generated for 7–23 individuals per population according to Vos et al. (1995) with some minor modifications (cf. Kropf et al. 2006; Kropf 2012): DNA was digested using the restriction enzymes *Mse*I and *Eco*RI. In the subsequent pre-selective amplification, primer pairs each containing one selective nucleotide were used. The selective amplifications were then performed with three primer combinations, with each primer containing two additional selective nucleotides (E + ACG/M + CGG [E37/M57], E + AGA/M + CTG [E39/M61], E + ATG/M + CGG [E45/M57]). These combinations have been applied successfully in previous studies (Kropf et al. 2003; Plenk et al. 2016, 2017) and were labelled by three different fluorescence dyes (E37: NED™, E39: 6-FAM™, E45: HEX™). For genotyping, the PCR products were mixed with an internal size standard (ROX™, ET550-R) and run on a MegaBACE DNA Analysis System (Amersham Biosciences, Freiburg, Germany). The raw data obtained was then aligned with the internal size standard using the program MegaBACE Fragment Profiler 1.2 (Amersham Biosciences) and a peak height threshold of 50 relative fluorescent units (RFUs) within a range of 60–550 base pairs fragment length. The generated presence/absence matrix of AFLP fragments was visually checked to exclude misinterpretations as described by Meudt and Clarke (2007). 54 individuals (i.e. six individuals, different on each plate) were replicated during lab work to test the reproducibility of fragments. Furthermore, blind samples were loaded to check runs for impurities (cf. Bonin et al. 2004).

For *cpDNA sequence analyses,* two regions, *rpL16* intron (primers: *rpL16F71*, *rpL16R1516*; Shaw et al. 2005) and *atpI*-*H* (primers: *atpI*, *atpH*; Shaw et al. 2007) were sequenced for six to nine individuals per population. Both plastid markers have been previously tested for our study species together with other cpDNA markers (i.e. *rpL32*, *rpS16, trnL*-*trnF*) and were selected due to their potential for higher intraspecific variation. The amplification was carried out under standard PCR conditions as described in Plenk et al. (2017). PCR-products (12 µl) were sent to LGC Genomics (Berlin, Germany) for cycle sequencing using the same primers. Alignment of cpDNA sequences obtained was done manually with BioEdit (version 7.2.5, Hall 1999).

### Data analyses

#### Performance parameters

Reproductive success was analysed for one panicle per mother plant using the following parameters: number of flowers, number of seeds, seed mass (g), fruit set (%), germination rate (%), and seedling survival rate (%). The *number of flowers* per panicle was determined as the total of mature seeds, undeveloped seeds and shed spikelets (i.e. multiplied by inherently six flowers per spikelet). The parameter *number of seeds* only includes the mature seeds counted in the respective panicle. However, as we must assume that shed spikelets contained mostly mature seeds, this parameter might be underestimated. To assess the magnitude of this possible influence on our results, we calculated the proportion of shed spikelets compared to the total number of spikelets, which was only 17.6%. The number of seeds was also used to calculate a mean weight per seed (i.e. *seed mass*) from the total seed mass of all mature seeds within a single panicle. *Fruit set* was calculated as the proportion of mature seeds from the number of flowers whereas the *germination rate* and *seedling survival rate* were determined as the proportion of germinated seeds/survived seedlings related to the number of seeds applied in the germination experiment. Since our data does not meet the assumptions of parametric analyses, we performed a non-parametric Kruskal–Wallis rank sum test (Kruskal and Wallis 1952) for regional comparison and a post hoc pairwise multiple comparison between regions (i.e. median differences) following Dunn’s test for stochastic dominance (Dunn 1964; implemented with the R-package ‘dunn.test’; Dinno 2017). Analyses were performed with the statistical program R (version 3.4.0; R Core Team 2017) and a significance level of α = 0.05.

#### AFLPs

Based on the complementary value of Nei and Li’s similarity coefficient (Nei and Li 1979), we performed an individual-based genetic distance analysis using PAUP* (version 4.0; Swofford 2002). Further, we computed a NeighborNet based on uncorrected P distances using Splits Tree4 (version 4.14.4; Huson and Bryant 2006). Bayesian Clustering was performed with BAPS (version 5.3; Corander et al. 2003, 2004) to reveal the most likely number of clusters (*K*) by means of stochastic optimisation. Clustering of individuals was performed with a maximum of *K *= 13 clusters and nine replicates for each *K*. We performed the analyses with and without geographical coordinates of populations as an informative prior. A Bayesian admixture analysis (Corander and Marttinen 2006) was calculated using the settings described in Plenk et al. (2016). Genetic differentiation among and within populations/regions was estimated in ARLEQUIN (version 3.5.2.2; Excoffier and Lischer 2010) using non-hierarchical and hierarchical analyses of molecular variance (AMOVA; cf. Plenk et al. 2017). These analyses were carried out for the whole dataset and for the (north)westernmost exclave and the Pannonian region, separately. To test for isolation by distance (Wright 1943; Hutchinson and Templeton 1999), pairwise *F*_ST_-values of the twelve *Poa badensis* populations were transformed to deal with linear sampling along our study transect (cf. Rousset 1997) and then regressed on geographical distance (i.e. km) among populations. Analyses were performed as described in Plenk et al. (2017) across the study transect and within regional subsets. Genetic (AFLP) diversity was estimated for the studied populations using the program POPGENE (version 1.31; Yeh et al. 1997) and the following diversity indices: Nei’s gene diversity (*H*_E_; Nei 1973), Shannon’s information index (*I*; Shannon and Weaver 1949), and percentage of polymorphic loci (*PLP*). To reduce potential unbalances caused by the unequal sample size of our AFLP data (ranging from 7–23 individuals per population; Table 1), we additionally carried out a rarefaction analysis for genetic diversity estimation (Petit et al. 1998) using AFLPdiv (version 1.1; Coart et al. 2005). Original AFLP band richness was adjusted to the minimum populational sample size within our dataset (i.e. n = 7). Genetic diversity was then calculated based on the number of AFLP phenotypes (i.e. band richness, *Br*), which are expected at each locus, for this adjusted sample size. Further, the percentage of polymorphic loci (*PLP*) was also calculated with this standardised sample size, whereby a locus is considered as polymorphic when *Br* ranged from 1.05 to 1.95 (5% level) or 1.01 to 1.99 (1% level; Coart et al. 2005).

#### cpDNA sequence data

Statistical parsimony haplotype networks were created with the program TCS (version 1.21, Clement et al. 2000) based on the combined alignment of both cpDNA sequences. Insertions/deletions which were longer than one base pair, as well as inversions, were coded as single-step mutations; (single) sequence gaps were treated as a fifth character state. Using Arlequin (ver. 3.5.2.2; Excoffier and Lischer 2010) genetic diversity indices, i.e. nucleotide [π] and haplotype diversity [*h*], were calculated based on cpDNA sequence variation. Testing deviation from neutrality, we further calculated Tajima’s D (Tajima 1989) to detect evidences for possible selection, recent expansion and/or genetic bottlenecks (cf. Christe et al. 2014).

## Results

### Performance parameters

In total, 1874 seeds from 223 mother plants were included in the germination experiment. On a regional scale, the number of mature seeds per panicle was highest in Central Hungary (mean 38.25 ± 40.28 SD; standard deviation) and lowest in Eastern Austria (mean 22.63 ± 31.05), differing significantly among regions (Kruskal–Wallis test: *p* = 0.004; Table 2). Results of the post hoc test demonstrated that the number of seeds differed significantly due to the low values observed in the Austrian region. The highest regional fruit set was found in Western Germany (mean 25.8% ± 20.4), whereas Eastern Austrian populations again showed the lowest value (mean 18.6% ± 17.4), differing significantly (Kruskal–Wallis test: *p* = 0.043). The mean seed mass per seed showed the highest values in the Hungarian (mean 0.3490 mg ± 0.4337) and the lowest in the Eastern Austrian region (mean 0.2899 mg ± 0.1990), demonstrating highly significant differences between regions (*p* < 0.001). In detail, seed mass was significantly different between the German and the Austrian, as well as the German and the Hungarian study region.

**Table 2:** Estimated reproductive success and differentiation of performance parameters among regions

Code	Est. PSC^a^	*N* performance analysis^b^	Number of flowers ± SD	Number of seeds ± SD	Fruit set ± SD	Seed mass g ± SD	Germination rate ± SD	Seedling survival rate ± SD
G1	1	18	110.30 ± 46.9245	13.95 ± 14.4749	0.1350 ± 0.1310	0.3372 ± 0.1508	0.8583 ± 0.1583	0.6470 ± 0.2813
G2	4	20	152.65 ± 85.4630	53.45 ± 55.0612	0.3234 ± 0.1996	0.3148 ± 0.0437	0.7465 ± 0.3398	0.5650 ± 0.2943
G3	3	20	108.75 ± 88.2090	36.05 ± 30.3531	0.3693 ± 0.2165	0.3250 ± 0.0772	0.8785 ± 0.1722	0.6808 ± 0.1873
G4	2	19	163.60 ± 70.6484	30.30 ± 31.1788	0.2051 ± 0.1814	0.3373 ± 0.1441	0.8311 ± 0.1563	0.6830 ± 0.2028
**Germany**	**2.5**		**133.83**	**33.44**	**0.2582**	**0.3286**	**0.8278**	**0.6434**
A1	1	19	100.10 ± 42.1687	24.85 ± 31.2499	0.2278 ± 0.2077	0.2839 ± 0.1192	0.8184 ± 0.2642	0.6154 ± 0.2469
A2	1	20	79.35 ± 36.6481	15.95 ± 13.4026	0.1969 ± 0.1351	0.2527 ± 0.0565	0.8265 ± 0.2106	0.6591 ± 0.2275
A3	3	14	99.80 ± 37.3358	9.35 ± 14.1357	0.0802 ± 0.1118	0.3625 ± 0.3484	0.8629 ± 0.2233	0.5226 ± 0.3230
A4	2	18	139.00 ± 64.9939	40.35 ± 45.6650	0.2375 ± 0.1889	0.2605 ± 0.1341	0.7894 ± 0.2145	0.4981 ± 0.2771
**Austria**	**1.75**		**104.56**	**22.63**	**0.1856**	**0.2899**	**0.8221**	**0.5797**
H1	3	20	174.65 ± 82.9212	44.70 ± 48.8360	0.2444 ± 0.2115	0.5003 ± 0.7832	0.7105 ± 0.3095	0.3478 ± 0.2296
H2	3	15	119.20 ± 72.5916	10.90 ± 13.8294	0.0906 ± 0.1250	0.2673 ± 0.3453	0.7907 ± 0.3171	0.4423 ± 0.3287
H3	4	20	167.75 ± 51.6607	64.20 ± 36.5436	0.3831 ± 0.2025	0.3125 ± 0.0367	0.7750 ± 0.1997	0.5038 ± 0.1679
H4	3	20	122.20 ± 45.0340	33.20 ± 35.5936	0.2568 ± 0.2202	0.3161 ± 0.1206	0.8215 ± 0.2515	0.5978 ± 0.2560
**Hungary**	**3.25**		**145.95**	**38.25**	**0.2437**	**0.3490**	**0.7733**	**0.4749**
**Differentiation among regions** (Kruskal–Wallis rank sum test)			***p < 0.001	**p = 0.004	*p = 0.043	***p < 0.001	n. s. *p* = 0.357	***p < 0.001
**Differentiation between pairs of regions** (post hoc Dunn’s test for stochastic dominance of median differences)			G–A*	G–A**	G–A**	G–A***	G–A n. s.	G–A n. s.
			G–H*	G–H n. s.	G–H n. s.	G–H**	G–H n. s.	G–H***
			A–H***	A–H**	A–H n. s.	A–H n. s.	A–H n. s.	A–H**

Performance parameters of *Poa badensis* populations studied across Central Europe and mean of regional performance in the three study regions Germany (G), Austria (A) and Hungary (H). Regional differences of performance parameters in the three study regions are given as *p*-values (based on Kruskal–Wallis tests); significant differentiation between regions is indicated with asterisks (*p ≤ 0.05, **p ≤ 0.01, ***p ≤ 0.001)

^a^Estimated population size category (PSC): 1: < 100, 2: 100–200, 3: 200–500, 4: 500–1000 individuals

^b^Number of included individuals,* SD* standard deviation

Overall, the level of germination rate after 41 days was high: i.e. 80.8% (± 24.1). The values varied only slightly between 87.9% (± 17.2; G3, Heidesheim) and 71.1% (± 31.0; H1, Budaörs). On regional level, germination was highest in the German exclave (mean 82.8% ± 22.4) and lowest in Central Hungary (mean 77.3% ± 26.8); albeit without statistical significance. A similar pattern was obtained for seedling survival with the regionally highest survival rate in Western Germany (mean 64.3% ± 24.5). The lowest survival rate was found in the Hungarian region (mean 47.5% ± 25.9); here, differences between regions were highly significant (Kruskal–Wallis test: *p *< 0.001) due to significant differences between the Austrian and Hungarian and the German and Hungarian region. Detailed results of all performance parameters are summarised in Table 2.

None of the performance parameters was significantly correlated with population size, although there was a slight trend to interdependence between fruit set, number of seeds and number of flowers. A significant positive correlation was shown for the seedling survival rate and geographical longitude (*r* = 0.657, *p* = 0.02), indicating gradually higher survival rates towards the (north)westernmost exclave. However, none of the other performance parameters was significantly correlated with this estimate of peripherality.

### Genetic structure

For each study region about 80 individuals (i.e. 241 in total) were included in the *AFLP analyses*, whereby for 30 of those samples the generation of reliable AFLP fragment patterns has failed. Therefore, subsequent statistical analyses were based on 211 individuals (Table 1) and 770 polymorphic AFLP fragments (i.e. 259, 217, and 295 of the three primer combinations, respectively; the latter count also including a single monomorphic fragment). In a *genetic distance analysis* individuals clustered predominantly according to their regional origin, with a high similarity between the Eastern Austrian and Central Hungarian populations and a stronger differentiation to and within the Western German populations. Within regions, grouping of individuals more or less reflected their respective population origin; nevertheless, particularly the population Hundsheimer Berg (A3) showed a heterogeneous composition (cf. Online Resource 2).

Testing the molecular variance (*AMOVA*) without regional structure, we found variation of 21.8% among and 78.2% within all populations (Online Resource 3). However, using a hierarchical approach based on the geographical origin (i.e. the three study regions), 11.0% of variation originated from the differentiation among regions, whereby 13.1% represented population differentiation within regions. The partitioning of data into two groups, representing the (north)westernmost exclave versus the Pannonian region (i.e. Austrian and Hungarian populations), revealed 12.3% of the variation among these groups and 14.1% among populations within groups. To test patterns of within-region population differentiation, we calculated two additional AMOVA analyses, i.e. one for the (north)westernmost exclave and one for the Pannonian region, resulting in 23.5% and 12.3% population differentiation, respectively (cf. Online Resource 3). The linear regression of pairwise transformed *F*_ST_-values and geographical distances between populations (i.e. km) revealed a significant isolation-by-distance pattern (Wright 1943) along our study transect (*r* = 0.4723; *p *= 0.0037). Within subsets we found significant isolation by distance in the Pannonian region (*r* = 0.3570; *p* = 0.0222), but not in the (north)westernmost exclave (*r* = − 0.2858; *p* = 0.4628).

*Bayesian clustering of individuals* revealed an optimal number of *K *= 3 groups within the whole dataset. Individuals of Austrian and Hungarian populations were again allocated together as one of these three groups, in the following called the Pannonian group/populations (only the populations Hundsheimer Berg, A3 and Csákvár, H4 showed some similarity to the German region). German individuals were differentiated into the two other groups, one containing the populations Eckelsheim (G1) and Weilersberg (G2; i.e. group G I) and the other one Heidesheim (G3) and Eierfels (G4; group G II; data not shown).

The *admixture analysis based on spatial clustering of individuals* (*K *= 3; Fig. 1) indicated low gene flow between the (north)westernmost exclave and the Pannonian region: i.e. less admixture in A3, H1, H2 and (almost) no admixture in G1, G2, G3, G4, A1, A2, A4, H3. Only the population Csákvár (H4) showed a comparatively high level of admixture with the German group G I (Fig. 1). The German populations Heidesheim (G3) and Eierfels (G4; i.e. both representing the group G II) demonstrated some admixture with the German group G I (Fig. 1).

**Fig. 2:**
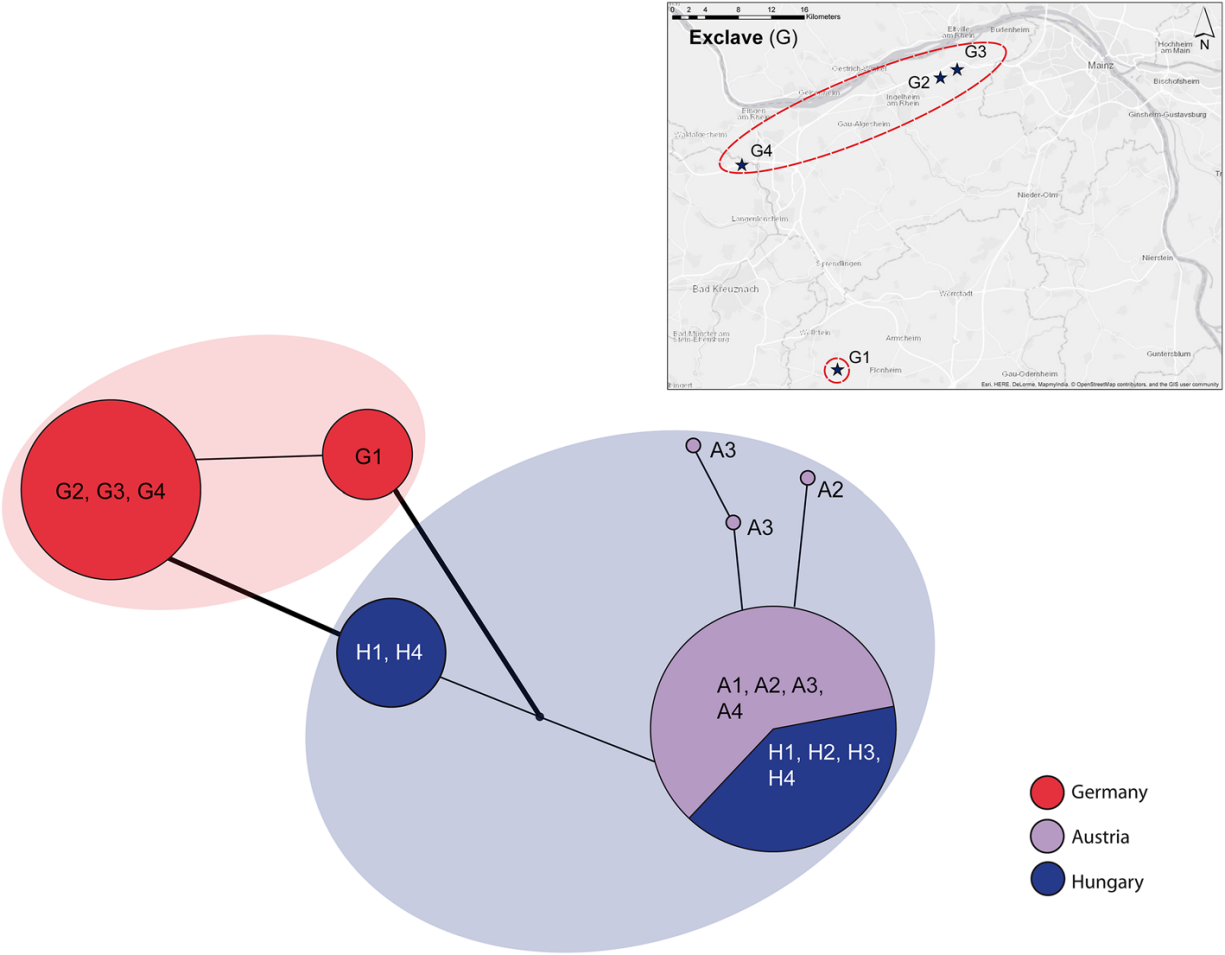
CpDNA haplotype network. TCS haplotype network showing the distinctiveness of the (north)westernmost exclave (G), separated from the Pannonian haplotype group (including Austrian (A) and Hungarian (H) populations) by one specific point mutation (at bp 883, *atpI*-*H*; bold lines). The inserted map illustrates the geographical isolation of population G1. For population abbreviations see Table 1

*Sequences from the chloroplast* (regions *rpL16, atpI*-*H*) were generated for 94 individuals, resulting in an alignment of 1507 base pairs length and seven haplotypes. We found two main haplotype groups, separated by one specific mutation at bp 883 (*atpI*-*H*): one German group and one Pannonian group (i.e. the latter containing individuals exclusively from Austria and Hungary). Within the German group individuals from the population Eckelsheim (G1) formed one specific haplotype, whereas the second haplotype in this group contained all other German populations (i.e. Weilersberg, G2; Heidesheim, G3 and Eierfels, G4). In contrast, the Pannonian group is characterised by further structuring (five haplotypes), but most of the individuals (i.e. 45 of 59 individuals; representatives of all Austrian and Hungarian populations) formed one major haplotype (see Fig. 2).

In addition to our main cpDNA dataset, we generated DNA sequences for the Mainzer Sand population (G5). The four individuals analysed were allocated to both haplotypes detected within the German exclave (see Fig. 2 and Online Resource 4).

### Genetic diversity

Different genetic diversity estimates based on our *AFLP data* basically produced similar results (Table 1). Comparing the diversity values (*H*_E_) of our studied populations with values compiled by Nybom (2004) for long-lived perennial species (0.25), occurring in a narrow geographical range (0.28), and characterised by an anemochorous seed dispersal (0.27), we found overall a comparatively low absolute genetic diversity in all populations and study regions. The absolute lowest (*H*_E_: 0.0457; *I*: 0.0755) and highest (*H*_E_: 0.1408; *I*: 0.2243) diversity values were found in the populations Eckelsheim (G1, population size category “PSC”: 1) and Pilisszentkereszt (H3, PSC: 4), respectively. Interestingly, the population Weilersberg (G2), showed the second lowest genetic diversity (*H*_E_: 0.0479; *I*: 0.0802), although being a large population (PSC: 4). After standardisation of AFLP band richness to the minimum populational sample size (i.e. rarefaction to 7, cf. Table 1), we revealed adjusted values for genetic diversity (*PLP*, *Br*). The estimates after rarefaction were slightly higher in some of the populations (Eckelsheim, G1; Eierfels, G4 and Eggendorf, A2; the latter one being the population with the originally highest number of individuals analysed). However, the highest and lowest values were again found in the populations Pilisszentkereszt (H3) and Eckelsheim (G1), respectively.

On regional average, genetic diversity values (*H*_E_, *I*) were highest in Central Hungary (*H*_E_: 0.1122; *I*: 0.1772) and lowest in Western Germany (*H*_E_: 0.0876; *I*: 0.1398). Genetic diversity of Eastern Austrian populations was only slightly higher (*H*_E_: 0.0906; *I*: 0.1438) than in the (north)westernmost exclave. However, the distribution of genetic diversity across regions varied slightly for *PLP*s: here, the lowest regional diversity was found in the Eastern Austrian study region and this pattern retained also after rarefaction.

Genetic diversity calculated from the main *cpDNA sequence data* showed overall very low diversity in the German region as each population (excluding G5) was monomorphic (Table 1). The highest population diversity was found in the populations Hundsheimer Berg (A3; haplotype diversity: 0.5238) and Csákvár (H4; nucleotide diversity: 0.0006). At the regional level, cpDNA haplotype diversity of the Austrian and Hungarian study region was similar (i.e. 0.40475 for Austria and 0.38095 for Hungary), while the Hungarian region showed the highest nucleotide diversity. Testing deviation from neutrality (i.e. Tajima’s D; Tajima, 1989) revealed no significant indications for departure from neutrality. None of the genetic diversity estimates was correlated with population size, geographical longitude (i.e. peripherality) or one of the performance parameters.

## Discussion

### Species’ performance and genetic composition along the study transect

By testing biogeographical hypotheses, e.g. a central-marginal gradient, we evaluated demographic characteristics (i.e. population density, population size), reproductive success and genetic composition along our study transect. Regarding the demographic characterisation of our studied populations, *Poa badensis* followed the hypothesised gradient (Sagarin and Gaines 2002; Abeli et al. 2014). In Germany, the species is classified as ‘vulnerable’ and regionally ‘endangered’, e.g. in Rhineland-Palatinate where only a few populations exist (see chapters below; Korneck et al. 1996). Although we have no concrete information on the total number of existing populations in Austria and Hungary, the species is more common there (Niklfeld 2013; Bartha et al. 2015). Hence, we conclude that the density of populations in our study regions is indeed declining towards the species (north)westernmost distribution limit. Moreover, we analysed the individual-richest populations in Hungary, represented by hundreds of individuals. The smallest populations within our study transect, most contained less than 200 individuals (Tables 1, 2), were found in Eastern Austria. However, the individual-poorest population analysed was again from Western Germany (i.e. Eckelsheim, n < 30).

On the contrary, our results did not indicate the expected reduced reproductive success of the (north)westernmost populations (cf. Abeli et al. 2014). The values of performance parameters were highest or at least equally high as values from the continuous part of the distribution range (i.e. Central Hungary; Table 2), as also described for *Stipa capillata* (Wagner et al. 2011b) and *Lychnis viscaria* (Lammi et al. 1999). Nevertheless, our performance parameters include some uncertainty, since they reflect the species’ reproductive success in situ (i.e. in the wild; cf. Wagner et al. 2011b) and may therefore include some maternal effects (Gutterman 2000).

Regarding the genetic composition of our study species, we observed a regional genetic impoverishment of (but see next Discussion chapter) and an increasing genetic differentiation among the (north)westernmost populations, as expected for a central-marginal gradient (Sagarin and Gaines 2002; Eckert et al. 2008; Sexton et al. 2009), but also partially for ‘stable’ rear edge populations (rather than for leading edge populations; Hampe and Petit 2005). Regional population differentiation was by far the highest in Germany. Pannonian populations from Austria and Hungary showed a lower population differentiation (Online Resource 3), which should also be found when further populations from the centre of the continuous Pannonian range are analysed, because gene flow is obviously high in this region. Both marker systems (AFLP & cpDNA) revealed a clear distinctiveness of the (north)westernmost exclave. Such a strong genetic distinctiveness and/or high population differentiation of peripheral populations has been observed, for example, in two other Poaceae species, *Stipa capillata* (Hensen et al. 2010; Wagner et al. 2011a) and *Stipa pennata* (Wagner et al. 2012), as well as in our previous study on *Linum flavum* (Plenk et al. 2017), which is based on a similar transect setting.

### Constitution of populations in the (north)westernmost isolated exclave

In the German exclave, the species’ reproductive success was not obviously reduced, while at the same time our analyses of genetic composition showed a partial genetic impoverishment. This pattern was somewhat surprising, as we did not expect to find reduced genetic diversity *and* high reproductive success in this region. The overall low level of genetic diversity observed in Western Germany, seems to be mainly a result of very low values found in two of the four investigated populations (i.e. Eckelsheim, G1, PLP % [7]: 21.7; Weilersberg, G2, PLP % [7]: 24.0). The populations Heidesheim (G3, PLP % [7]: 49.7) and Eierfels (G4, PLP % [7]: 44.7) did not show reduced levels of genetic diversity; the values observed there are comparable to those of the continuous distribution in Central Hungary (PLP  % [7]: 41.1; see Table 1). Hensen and Wesche (2007) observed comparatively high levels of genetic diversity (PLP %: 50.0–74.2; genetic diversity: 0.20–0.29) in Central German populations of *Poa badensis*, but a moderate to low genetic differentiation among populations. Compared to other thermophilic steppe species from the same plant family (i.e. *Stipa capillata*, Hensen et al. 2010; *Stipa pennata* agg., Wagner et al. 2012; Durka et al. 2013), or other steppe species occurring in calcareous rock/sand vegetation (i.e. *Globularia bisnagarica*, Honnay et al. 2007; *Alyssum montanum*, Španiel et al. 2012), these values represent high levels of genetic diversity at the (north)westernmost border of their distribution. Consequently, Hensen and Wesche (2007) described the species as only slightly affected by fragmentation and assessed genetic exchange between populations in their study region as still possible. In our more western German study region, *P. badensis* is obviously affected by reduced gene flow (cf. Figures 1, 2), which is also clearly demonstrated by the strong genetic within-region population differentiation (Online Resource 3). Although populations of wind-pollinated and -dispersed species in general are assumed to have a greater potential for genetic exchange, the low dispersal capacity of our study species together with the scattered availability of suitable habitats in our study region have led to this increased genetic differentiation. In addition, we found no clear evidence for isolation by distance, possibly indicating genetic drift already occurring in the Western German populations.

Even though the regional genetic diversity is lowest in the German exclave, we basically interpret its comparatively high reproductive success as indicator for good reproductive capability. As we obtained indications of different ploidy levels within our study transect (i.e. diploid populations in Austria and Hungary, but tetraploid populations in Germany, Online Resource 1), a possible explanation for the high performance parameters observed in the (north)westernmost exclave might be the occurrence of one (or more) polyploidization events, likely occurring during warm (postglacial) stages. This may have led to a stronger reproductive potential, probably as an evolutionary strategy to overcome the more unfavorable conditions at the absolute range limit. Moreover, polyploid populations may also show generally higher levels of genetic diversity (cf. *Alyssum montanum*, Španiel et al. 2011).

However, the species’ biogeographical history and specific traits (e.g. longevity) may have aided to retain a certain level of genetic diversity, as observed in the populations Heidesheim (G3) and Eierfels (G4). On the contrary, the populations Eckelsheim (G1) and Weilersberg (G2) are likely less viable and show low genetic diversity, which is driven by their histories. Although population size did not correlate with the genetic diversity in general, at least one of these two populations is characterised by small population size (G1, less than 30 individuals). Nevertheless, low genetic diversity of populations does not necessarily imply that they have to be less viable. Habel and Schmitt (2012, 2018) argued that specialised species, as a consequence of habitat loss and degradation, are commonly characterised by such low levels of genetic diversity accompanied by high genetic distinctiveness. However, they may persist in small-sized remnants of suitable habitats over long periods of time, just slightly affected by population bottlenecks and inbreeding due to their limited, but locally adapted genetic composition.

### Regional biogeography and conservation history of the German exclave

Ellenberg and Leuschner (2010) quote *Poa badensis* among other steppe species as a relict, surviving on sandy/rocky grassland sites in Central Europe since the last glacial. However, they also remarked that most of the populations occurring today are likely younger (i.e. originating from Neolithic Age or early mediaeval times; cf. Willis et al. 1998). In Germany, relict localities of thermophilic species are described from dry regions like the Unstruttal (Becker 1999), the (Lower) Nahe region, aeolian deposits of large rivers (e.g. the north and east of the Rhinehessian plateau; Blaufuss and Reichert 1992; Witschel 1991) or south-exposed rock faces of low mountain ranges (e.g. the Swabian Alb; Wilmanns 2005). Our German study region is situated in two of these refugial areas, the Rhinehessian plateau and the adjacent Lower Nahe region. They are characterised by calcareous aeolian sands, deposited along the northern slopes of the plateau in glacial and early postglacial times (Jännicke 1892; Ellenberg and Leuschner 2010), as well as by the basic conglomerate of the so-called Waderner Schichten of the Upper Rotliegend Group (Weiss 1889).

In our study, we included four of the five mentioned populations from Rhineland-Palatinate by performing all analyses, which overall characterised the (north)westernmost exclave as genetically impoverished. However, there are two populations, Eierfels (G4) and Heidesheim (G3), showing comparatively high levels of genetic diversity. As their history is very different, we will go into more detail. The population Eierfels (G4) is situated on a flat ridge of basic conglomerate in the Lower Nahe region which is protected as natural monument and harbours a unique xerothermic vegetation (Korneck 1998; Oesau and Frahm 2007). The population of *P. badensis* is rather small, but likely represents an old occurrence probably existing there since the early postglacial (cf. Blaufuss and Reichert 1992 and citation therein). In Heidesheim (G3) our study species grows secondary on a cemetery wall. This rather large and likewise old population persists there over a long period of time and represents one of the two still existing populations on the northern edge of the Rhinehessian plateau (i.e. beside the relict population in the Mainzer Sand). It seems possible that the species had arrived there during the construction of the wall with limestone mortar or, shortly after completion, via natural dispersal (e.g. by wind) from the close surrounding area, where the species previously occurred more frequently (cf. Blaufuss and Reichert 1992; Hodvina and Cezanne 2008).

Occurring in the close vicinity of Heidesheim (i.e. only 1.5 km apart), the population Weilersberg (G2) is characterised by a different and considerably younger history. This area was originally used as a sandpit and later designated as a nature reserve. To our knowledge, *P. badensis* had been introduced to this site by taking (seed) material from the closely located population Heidesheim (G3). However, in our AFLP data we found the population Weilersberg grouping together with Eckelsheim and not with the assumed source of the seed transfer (Fig. 1). Therefore, we may assume that possibly a second gene pool has been used during the introduction, or that based on a heterogeneous source, lineages were sorted out differently given population bottlenecks. Considering this specific history, the extremely low genetic diversity observed in this large population is not surprising and likely reflects this artificial (recent) founder event. However, the reproductive success in general was not significantly reduced by this bottleneck. Geographically most isolated from the other occurrences of *P. badensis* in Western Germany, the population Eckelsheim (G1) is again located on a rocky outcrop belonging to the Waderner Schichten as part of the Rhinehessian Plateau (Fig. 1). This isolated location is also reflected in our cpDNA results, where Eckelsheim represents one distinct haplotype within the German haplotype group (Fig. 2). The estimated genetic diversity of this population was by far the absolute lowest from all analysed populations, which we attribute to the obvious lack of gene flow and the very low population size (Table 1). Nevertheless, in combination with the not reduced reproductive success of this population, an adaptation to low genetic diversity as described by Habel and Schmitt (2012; 2018) might be assumed.

Our AFLP data revealed admixture between the populations Heidesheim (G3) and Eierfels (G4; together representing the group GII, Fig. 1) and the German group GI. Due to small population size (Eckelsheim, G1) and recent translocation (Weilersberg, G2) the other two German populations missed such an admixture pattern. To a lesser extent we also found admixture between the German group GI and the Pannonian region, indicating an earlier genetic connection of the (north)westernmost exclave with the continuous Pannonian occurrences. Despite that, we observed that 123 (30.4%) of 404 regionally unique AFLP fragments (cf. Petit et al. 1998) were only present in the (north)westernmost exclave. Within the continuous Pannonian distribution area, 281 (69.6%) of these regionally unique AFLP fragments were found. In this context, having nearly one-third of unique AFLP fragments, the peripheral populations in Western Germany represent a considerable and unique part of the total species’ genetic variation (cf. Plenk et al. 2017). Moreover, we obtained evidence for the relict status and high conservation relevance of the Mainzer Sand population (G5) although it was represented by just four individual plants. The four individuals analysed were allocated to both detected haplotypes of the German exclave (cf. Figure 2 and Online Resource 4), demonstrating that this probably oldest relict population (Korneck and Pretscher 1984) might still hold the full spectrum of genetic diversity original to this region.

Therefore, we interpret the observed genetic composition, demographic traits and reproductive success within the German exclave as evidence of a long-term (successful) in situ survival, probably since early postglacial times, which we especially hypothesise for the populations Eckelsheim (G1), Eierfels (G4), and Mainzer Sand (G5). We moreover conclude, that the populations of the Waderner Schichten (e.g. Eierfels, Eckelsheim) may originate from the probably slightly earlier populated occurrences along the northern edge of the Rhinehessian plateau (e.g. Mainzer Sand and other previously existing ones) and spread there along the river systems of Nahe and subsidiary streams to the west and south (cf. Blaufuss and Reichert 1992; Hodvina and Cezanne 2008).

Nevertheless, we also have to consider that the species may have reached suitable sites in Western Germany more recently via a long-distance dispersal (LDD) event. In this case, we would expect low genetic diversity and less genetic differentiation of these founder populations from a potential source in more central/eastern areas (cf. Wróblewska 2008). Although we observed some admixture between the German and Pannonian region, our data clearly showed a genetic distinctiveness of the (north)westernmost exclave in both marker systems (cf. Figures 1, 2). We, therefore, concluded that the populations of the German exclave do not represent descendants of a recent LDD-event. However, analysing additional populations of *P. badensis* from the few existing occurrences “linking” the German exclave and the Pannonian region may strengthen this conclusion.

Due to their rarity, peripheral populations are often under protection, although they are generally considered as genetically impoverished and less competitive (Lesica and Allendorf 1995). With our study on *P. badensis,* we could clearly demonstrate that these assumptions are not applicable in general. However, due to the low abundance and the lack of connectivity, populations might be nevertheless under pressure if they are not (yet) adapted to low levels of genetic diversity (Habel and Schmitt 2012; 2018). Therefore, we must also consider a possible time lag (Lindborg and Eriksson 2004) between the decline of population sizes and the species’ reproductive success and genetic diversity (cf. Kropf 2012).

## Electronic supplementary material

Below is the link to the electronic supplementary material.

Supplementary material 1 (PDF 202 kb)

Supplementary material 2 (PDF 2609 kb)

Supplementary material 3 (PDF 96 kb)

Supplementary material 4 (PDF 778 kb)
