# Supplementary material for: Long-term survival and successful conservation? Low genetic diversity but no evidence for reduced reproductive success at the north-westernmost range edge of Poa badensis (Poaceae) in Central Europe
Source: Biodivers Conserv. 2019 Feb 27;28(5):1245–65. doi: 10.1007/s10531-019-01722-x (PMC6399750; doi:10.1007/s10531-019-01722-x)
Supplement: Supplementary file 1 — Supplementary material 1 (PDF 202 kb) [file 10531_2019_1722_MOESM1_ESM.pdf]

Long-term survival and successful conservation? Low genetic diversity but no evidence for reduced reproductive success at the north-westernmost range edge of *Poa badensis* (Poaceae) in Central Europe.

Biodiversity and Conservation. Plenk K, Bardy K, Höhn M & Kropf M.

Corresponding author: Kristina Plenk; Institute for Integrative Nature Conservation Research, University of Natural Resources and Life Sciences, Vienna, Gregor-Mendel-Str. 33, 1180 Vienna, Austria; kristina.plenk@boku.ac.at

Online Resource 1 Exemplarily tested ploidy levels of *Poa badensis* individuals representing the three study regions (G, A, H)

The tested Austrian and Hungarian individuals showed on average a peak at 85.60 (FL1; first peak), those from the German exclave at 162.02 (FL1; first peak), i.e. indicating different ploidy levels taken as “diploidy” and “tetraploidy”. In both graphs the second peak represents the used standard *Pisum sativum* ‘Kleine Rheinländerin’ (FL1: 351.92).

**“diploid”**	**“tetraploid”**
Austria (A), Hungary (H)	Germany (G)

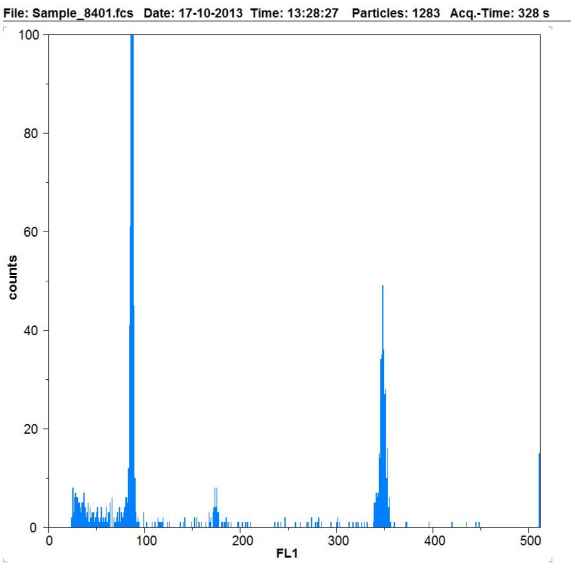

sample: A2 SEG 10_9

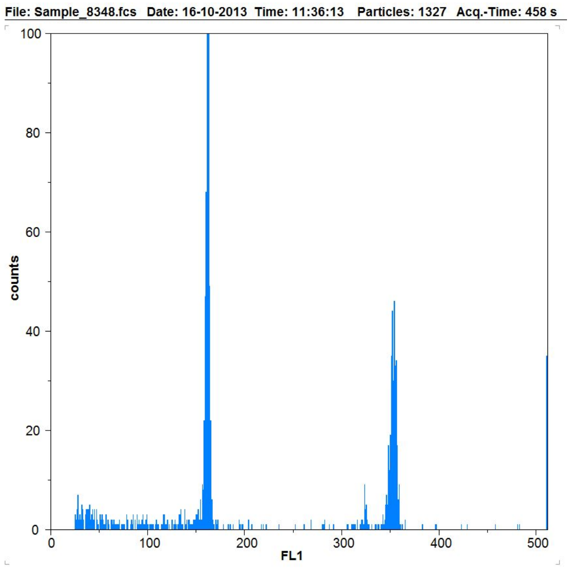

sample: G1 ECK 3_5

	Mean-x	CV-x %		Mean-x	CV-x %
A1 FAB 2_1	83.88	1.62	G1 ECK 3_5	162.06	1.08
A2 SEG 10_9	87.06	2.00	G1 ECK 7_2	161.84	1.22
H1 BDS 9_9	87.29	2.30	G2 WLB 2_8	161.69	1.65
H2 VPA 12_7	84.18	1.73	G2 WLB 9_5	162.49	1.49
mean	**85.60**		mean	**162.02**	
