# Supplementary material for: Long-term survival and successful conservation? Low genetic diversity but no evidence for reduced reproductive success at the north-westernmost range edge of Poa badensis (Poaceae) in Central Europe
Source: Biodivers Conserv. 2019 Feb 27;28(5):1245–65. doi: 10.1007/s10531-019-01722-x (PMC6399750; doi:10.1007/s10531-019-01722-x)

Long-term survival and successful conservation? Low genetic diversity but no evidence for reduced reproductive success at the north-westernmost range edge of *Poa badensis* (Poaceae) in Central Europe.

Biodiversity and Conservation. Plenk K, Bardy K, Höhn M & Kropf M.

Corresponding author: Kristina Plenk; Institute for Integrative Nature Conservation Research, University of Natural Resources and Life Sciences, Vienna, Gregor-Mendel-Str. 33, 1180 Vienna, Austria; kristina.plenk@boku.ac.at

Online Resource 2 Individual-based Neighbor-Net of *Poa badensis* (AFLP data)

The individual-based Neighbor-Net represents the three study regions, marked with 'A', 'H', and 'GI'/'GII', i.e. representing the Austrian and Hungarian individuals and the two German subgroups (cf. Figure 1).

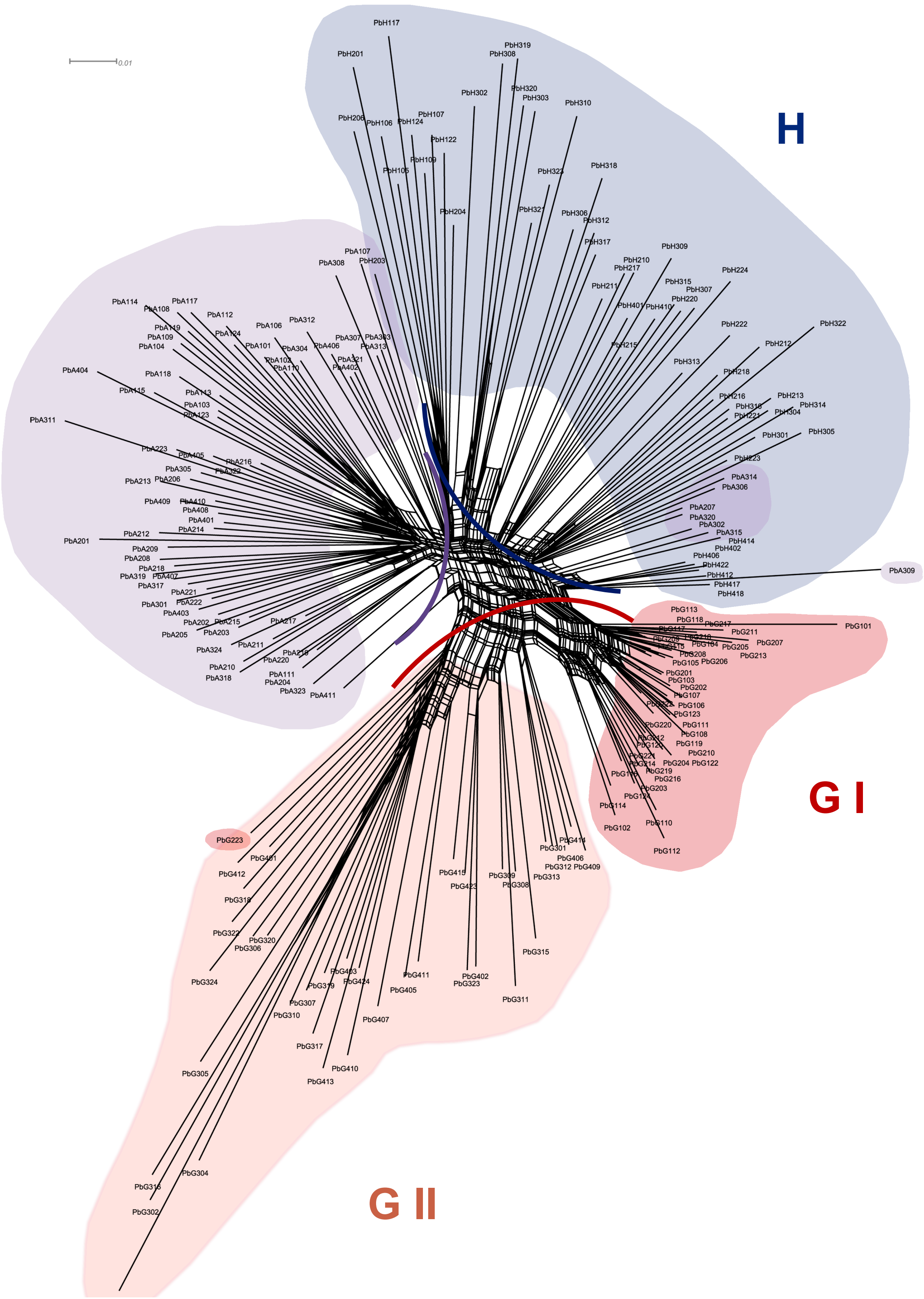

Supplement: Supplementary file 2 — Supplementary material 2 (PDF 2609 kb) [file 10531_2019_1722_MOESM2_ESM.pdf]
