# Supplementary material for: Long-term survival and successful conservation? Low genetic diversity but no evidence for reduced reproductive success at the north-westernmost range edge of Poa badensis (Poaceae) in Central Europe
Source: Biodivers Conserv. 2019 Feb 27;28(5):1245–65. doi: 10.1007/s10531-019-01722-x (PMC6399750; doi:10.1007/s10531-019-01722-x)
Supplement: Supplementary file 3 — Supplementary material 3 (PDF 96 kb) [file 10531_2019_1722_MOESM3_ESM.pdf]

Long-term survival and successful conservation? Low genetic diversity but no evidence for reduced reproductive success at the north-westernmost range edge of *Poa badensis* (Poaceae) in Central Europe.

Biodiversity and Conservation. Plenk K, Bardy K, Höhn M & Kropf M.

Corresponding author: Kristina Plenk; Institute for Integrative Nature Conservation Research, University of Natural Resources and Life Sciences, Vienna, Gregor-Mendel-Str. 33, 1180 Vienna, Austria; kristina.plenk@boku.ac.at

Online Resource 3 AMOVA analyses (AFLP data)

Analyses of molecular variance carried out without regional structure, as well as for the three geographical regions and the (north)westernmost exclave vs. the Pannonian region (i.e. Austrian and Hungarian populations). Further, non-hierarchical analyses were calculated to obtain population differentiation within the (north)westernmost exclave and the Pannonian region, respectively. Significance levels: * $p < 0.05$; ** $p < 0.01$; *** $p < 0.001$.

Source of variation	*d.f.*	*Sum of squares*	*Variance components*	*% of total variance*
**Test without regional structure (all populations analysed)**				
Among all populations	11	2521.491	10.90166	21.76***
Within populations	199	7800.097	39.19647	78.24
**Hierarchical analysis between the three geographical regions**				
Among regions	2	1125.476	5.70280	11.04**
Among populations within regions	9	1396.014	6.77024	13.10***
Within populations	199	7800.097	39.19647	75.86***
**Hierarchical analysis between the (north)westernmost exclave and the Pannonian region**				
Among groups	1	840.328	6.54370	12.29**
Among populations within groups	10	1681.162	7.49541	14.08***
Within populations	199	7800.097	39.19647	73.63***
**Subset:** *population differentiation within the (north)westernmost exclave*				
Among populations	3	736.508	10.49332	23.54***
Within populations	77	2624.282	34.08159	76.46
**Subset:** *population differentiation within the Pannonian region*				
Among populations	7	964.192	5.97509	12.32***
Within populations	122	5187.585	42.52118	87.68
