# Supplementary material for: Long-term survival and successful conservation? Low genetic diversity but no evidence for reduced reproductive success at the north-westernmost range edge of Poa badensis (Poaceae) in Central Europe
Source: Biodivers Conserv. 2019 Feb 27;28(5):1245–65. doi: 10.1007/s10531-019-01722-x (PMC6399750; doi:10.1007/s10531-019-01722-x)

Long-term survival and successful conservation? Low genetic diversity but no evidence for reduced reproductive success at the north-westernmost range edge of *Poa badensis* (Poaceae) in Central Europe.

Biodiversity and Conservation. Plenk K, Bardy K, Höhn M & Kropf M.

Corresponding author: Kristina Plenk; Institute for Integrative Nature Conservation Research, University of Natural Resources and Life Sciences, Vienna, Gregor-Mendel-Str. 33, 1180 Vienna, Austria; kristina.plenk@boku.ac.at

Online Resource 4 CpDNA haplotype network, including additional individuals from the Mainzer Sand

In this analysis four individuals from the fifth German population, Mainzer Sand (G5), completed the original dataset. The resulting TCS haplotype network basically remained unchanged: 7 haplotypes and a differentiation of the (north)westernmost exclave (shaded in red) from the Pannonian haplotype group at one specific mutation point could again be observed (cf. Figure 2). However, the four individuals from the Mainzer Sand (G5) were allocated to both haplotypes of the German exclave, demonstrating that this probably oldest relict population still holds the full spectrum of genetic diversity characterising this region (see also inserted map). For population abbreviations see Table 1.

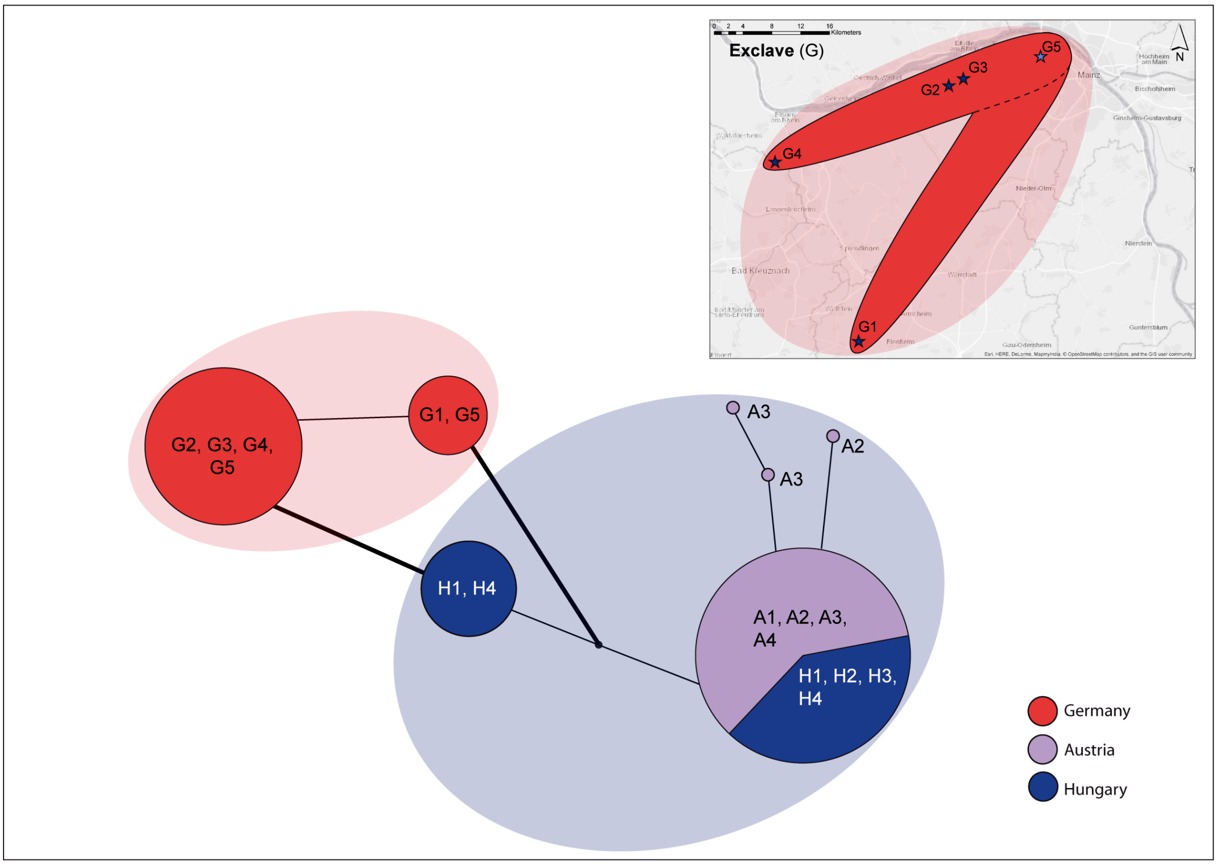

Supplement: Supplementary file 4 — Supplementary material 4 (PDF 778 kb) [file 10531_2019_1722_MOESM4_ESM.pdf]
